# Early Enteral Nutrition and Surgical Outcomes After Repair of Penetrating Bowel Injuries: A Retrospective Cohort Study From the Yemeni Civil War

**DOI:** 10.7759/cureus.112467

**Published:** 2026-07-11

**Authors:** Abdulkafi M Shamsan, Zaki N Nasr, Basma M Alkholidy, Hamdah A ALomeri

**Affiliations:** 1 General Surgery, Taiz University, Taiz, YEM; 2 Surgery, Taiz University, Taiz, YEM

**Keywords:** early enteral nutrition, penetrating bowel injuries, surgical site infection, trauma laparotomy, war surgery

## Abstract

Introduction: Penetrating bowel injuries (PBIs) are the most common serious consequence of abdominal gunshot and blast injuries in conflict zones and remain associated with substantial morbidity. Although international perioperative guidelines endorse early enteral nutrition (EEN), its safety and effectiveness after operative repair of PBIs in resource-limited, war-affected hospitals remain poorly established. We aimed to evaluate the association between the timing of postoperative enteral nutrition and predefined outcomes in adults undergoing operative repair of conflict-related PBIs, including postoperative complications, gastrointestinal recovery, hospital resource utilization, and mortality.

Methods: This was a retrospective single-center cohort study conducted at Al-Safwa Hospital, Taiz, Yemen, between January 2017 and December 2023. Adult patients (≥18 years) with intraoperatively confirmed PBIs who underwent primary repair or resection with anastomosis were included. Patients were stratified by timing of postoperative enteral nutrition into EEN (less than 48 hours) and delayed enteral nutrition (DEN) (more than 48 hours). Primary outcomes included postoperative complications occurring within 30 days, namely, surgical site infection (SSI), anastomotic leak, enterocutaneous fistula, fascial dehiscence, intra-abdominal abscess, pneumonia, sepsis, acute kidney injury (AKI), and mortality; secondary outcomes were gastrointestinal recovery, hospital and intensive care unit (ICU) length of stay, and reoperation. Group comparison used Mann-Whitney U, χ², or Fisher’s exact tests. Multivariable logistic regression identified independent predictors of complications.

Results: Of 109 included patients, the median age was 28 years; men constituted n = 102/109 (93.6%). The most common mechanism of gunshot wounds is n = 91/109 (83.5%). Cases who received EEN were n = 94 (86.2%), and those who received DEN were n = 15 (13.8%). EEN was associated with earlier return of bowel sounds (1 vs. 2 days, p = 0.021), lower overall complication rate (n = 20/94, 21.3% vs. n = 9/15, 60.0%; p = 0.003), lower SSI (n = 3/94, 3.2% vs. n = 7/15, 46.7%; p < 0.001), and no mortality (0/94, 0% vs. n = 3/15, 20%; p = 0.002). In an exploratory multivariable logistic regression model adjusted for high American Association for the Surgery of Trauma (AAST) grade, peritoneal contamination, and associated injuries, DEN remained associated with higher odds of postoperative complications (adjusted odds ratio (OR) 3.98, 95% confidence interval (CI) 1.20-13.23; p = 0.024). This estimate should be interpreted cautiously because of the small DEN group and the limited number of events.

Conclusion: In this conflict-zone cohort, EEN within 48 hours after PBI repair was feasible and was associated with faster gastrointestinal recovery, fewer postoperative complications, shorter hospital and ICU stays, and lower mortality than delayed feeding. These observational findings support considering EEN when clinically feasible after adequate source control and stabilization, while underscoring the need for prospective multicenter studies with standardized feeding protocols.

## Introduction

Penetrating abdominal trauma (PAT) accounts for a substantial proportion of casualties in armed conflict, with the small bowel being the most frequently injured viscus and gunshot or blast-related shrapnel wounds being responsible for the majority of cases [1,2]. Although overall mortality from war-related abdominal trauma has decreased over successive conflicts, penetrating bowel injuries (PBIs) remain associated with significant morbidity related to peritoneal contamination, infectious complications, and impaired anastomotic healing [1,2].

Surgical repair is the cornerstone of management of PBIs, but postoperative recovery is shaped by additional factors, particularly the metabolic stress response and nutritional status of trauma patients [3]. Patients with PBI, although often young and previously healthy, develop a hypercatabolic state that accelerates protein breakdown, impairs immune function, and predisposes them to infection and delayed wound healing [3,4].

Traditional postoperative management after intestinal repair has historically relied on prolonged fasting, nasogastric decompression, and delayed oral intake, motivated by concerns regarding anastomotic integrity [5]. Contemporary evidence has reversed this paradigm: early enteral nutrition (EEN), generally defined as initiation of enteral feeding within 48 hours after surgery, preserves gut mucosal integrity, attenuates bacterial translocation, and supports collagen deposition at anastomoses [4,6-9]. EEN is now embedded in Enhanced Recovery After Surgery (ERAS) and Western trauma protocols [4,10,11].

Despite this evidence base, delayed enteral nutrition (DEN) is still common in low- and middle-income countries and in conflict-affected settings, where surgeons must operate in austere environments with intermittent supplies, complex injuries, and limited high-level evidence specifically applicable to their patients [12]. Therefore, we conducted a retrospective cohort study at a tertiary trauma referral hospital in Taiz, Yemen, to compare postoperative outcomes between patients receiving EEN within 48 hours and those receiving DEN after 48 hours following operative repair of PBIs. The primary objective was to compare the occurrence of predefined 30-day postoperative complications, including infectious, anastomotic, wound, systemic, and mortality outcomes. Secondary objectives were to compare gastrointestinal recovery, hospital and intensive care unit (ICU) resource utilization, reoperation, and readmission. Given the observational design, the study was designed to evaluate associations rather than establish causality.

## Materials and methods

Study design, setting, and period

This was a retrospective single-center cohort study conducted at Al-Safwa Hospital, a tertiary referral hospital and major trauma center for penetrating injuries in Taiz, Yemen. The study period was from January 1, 2017, to December 31, 2023.

Population and eligibility criteria

During the study period, a total of 450 patients were admitted with PAT. Of 450 PAT patients screened, 341 were excluded; 310 had only solid organ injury and abdominal wall injury, 20 patients were under 18 years, and 11 died within 24 hours. The remaining 109 patients confirmed intraoperatively with PBIs who underwent surgical repair and received postoperative enteral nutrition were included. Inclusion criteria were adult patients aged ≥18 years, with confirmed PBI, surgical repair, survival ≥ 24 hours, receipt of enteral nutrition, and complete medical records. Patients were excluded if they were younger than 18 years or had blunt trauma, isolated solid organ injury, non-operative management, incomplete records, or death within 24 hours. Patients who died within 24 hours of admission or operation were excluded because they did not have a meaningful opportunity to receive postoperative enteral nutrition or to be classified reliably into the EEN and DEN exposure groups. Early deaths in this setting were also more likely to reflect overwhelming initial injury severity, exsanguination, or irreversible physiologic derangement rather than postoperative nutritional strategy. We recognize that this exclusion may introduce survival and selection bias by removing the sickest patients; this issue is explicitly addressed in the Limitations section.

Data collection and variables

Data were collected from medical records, operative notes, and nursing charts using a standardized form (Appendix). Variables collected included demographic data, injury characteristics, surgical management, and nutritional variables. Primary outcomes (within 30 days of surgery or during the index admission) were surgical site infection (SSI), anastomotic leak, enterocutaneous fistula, fascial dehiscence, intra-abdominal abscess, pneumonia, sepsis, acute kidney injury (AKI), and mortality. Secondary outcomes were time to first bowel sounds, time to first flatus, hospital length of stay (HLOS), ICU admission and ICU length of stay, reoperation, and 30-day readmission. Data abstraction was performed independently by three authors using a standardized form, with discrepancies resolved by consensus.

Exposure groups and definition

EEN was defined as initiation of any enteral intake, oral or tube-based, within 48 hours after surgery. DEN was defined as the initiation of enteral intake after 48 hours. The 48-hour threshold was chosen because it is commonly used in perioperative and critical-care nutrition literature, is consistent with ERAS-oriented postoperative feeding principles [13], and is a pragmatic cutoff in resource-limited conflict settings where commercial formulas, laboratory monitoring, and ICU resources may be intermittently available. Exposure definition was based on the first documented postoperative day of enteral intake in nursing or physician notes. Shock on admission was defined as systolic blood pressure < 90 mmHg at initial presentation. Bowel injuries were graded retrospectively using the American Association for the Surgery of Trauma (AAST) Organ Injury Scale [14] based on operative-note descriptions. The mechanism of injury was classified as a gunshot wound, blast-related shrapnel injury, or other.

Surgical and nutritional protocols in conflict settings

All patients received empiric broad-spectrum intravenous antibiotics before or within the first hour of laparotomy incision, targeting enteric gram-negative and anaerobic organisms. Because antibiotic availability varied during the conflict period, the specific regimen and duration were not completely standardized and were individualized according to injury severity, degree of contamination, associated injuries, source control, and clinical evolution. Antibiotic regimen and duration were therefore not included as comparative exposure variables in the present analysis. Operative management was individualized according to bowel segment, injury grade, tissue viability, hemodynamic status, degree of peritoneal contamination, associated injuries, and surgeon judgment. Primary repair was generally favored for localized low-grade injuries with viable bowel edges and limited tissue loss. Resection with anastomosis was used for devitalized bowel, transection, multiple destructive injuries, or segmental loss when the surgeon judged anastomosis to be safe. Stoma formation was reserved for selected patients with severe contamination, physiologic instability, destructive distal colorectal injury, or concern regarding anastomotic safety. Stapling devices were not used because of resource limitations; repairs and anastomoses were handsewn. Most of the bowel injury repairs were managed by board-certified general surgeons with Arab Board of Health specialization (AGSB) or equivalent fellowship qualifications. In contrast, a limited number were managed by MD-certified surgeons who held a medical doctorate with additional surgical training but without subspecialty certification. All procedures were performed with the assistance of surgical residents from our surgical department. No standardized written feeding protocol existed during the study period. In routine practice, feeding initiation was determined by the attending surgeon's judgment following a bedside assessment of clinical stability and anticipated tolerance. Typical factors considered included adequate operative source control, hemodynamic stability, absence of uncontrolled vomiting or progressive abdominal distention, and no clinical suspicion of ongoing peritonitis, missed injury, or anastomotic/suture-line leak. Return of bowel sounds was recorded as a recovery outcome but was not used as the sole mandatory trigger for feeding. Initial feeding usually consisted of clear fluids or oral rehydration solution when available, followed by gradual advancement to locally prepared soft diets such as soup and rice as tolerated. Feeding was slowed, withheld, or reassessed if vomiting, worsening distention, peritonitis, or other signs of intolerance developed.

Statistical analysis

Statistical analysis was performed using the Statistical Package for Social Sciences (SPSS), 24th edition software (IBM Corp., Armonk, NY, USA). Descriptive statistics were calculated for all variables. Categorical variables were presented as frequencies and percentages. Continuous variables were presented as median and interquartile range (IQR) due to non-normal distribution, confirmed by the Shapiro-Wilk test. Group comparisons used the Mann-Whitney U test for continuous variables and chi-squared or Fisher’s exact tests for categorical variables, as appropriate. Odds ratios (ORs) with 95% confidence intervals (CIs) for binary outcomes. A two-tailed p-value < 0.05 was considered statistically significant. Logistic regression was performed to identify independent predictors of postoperative complications. Variables with p < 0.20 in univariate analysis or clinical relevance were included in the final model, which included DEN (>48 hours), presence of associated injuries, peritoneal contamination, and high AAST grade (III-V). Because this was a retrospective cohort including all eligible patients during the study period, no a priori sample-size calculation was performed. The analysis should therefore be interpreted as exploratory and hypothesis-generating. Precision was limited by the small DEN group and by the limited number of postoperative complication events, particularly for rare outcomes and outcomes with zero events in one group. To reduce overfitting, the multivariable model was restricted to variables selected by univariate association or strong clinical relevance; however, adjusted ORs should be interpreted cautiously because of the limited event count and wide CIs.

## Results

Patient and injury characteristics

Of 450 PAT records screened, 109 patients with operatively confirmed PBI met the eligibility criteria. The median age was 28 years (IQR 22-35); 102 patients (93.6%) were male, giving a male-to-female ratio of approximately 14.6:1. Pre-existing comorbidities were uncommon (n = 6/109, 5.5%), with diabetes mellitus the most frequent. Gunshot wounds were the predominant mechanism (n = 91/109, 83.5%), followed by blast-related shrapnel injuries (n = 16/109, 14.7%) and other mechanisms (n = 2/109, 1.8%). Almost all patients (98.2%) reached the operating room within six hours of injury. The injury patterns and surgical management are summarized in Table 1.

**Table 1:** Injury patterns, AAST severity, and surgical management in 109 patients with penetrating bowel injuries. Percentages are calculated within each subgroup as defined in the body rows. AAST classification reflects injuries, while surgical data represent procedures performed. AAST: American Association for the Surgery of Trauma Organ Injury Scale

Variable	n	%
Injury pattern		
Isolated small-bowel injury	56	51.4
Isolated colon injury	31	28.4
Combined small-bowel and colon injury	18	16.5
Rectal injury	4	3.7
Mechanism of injury		
Gunshot wound	91	83.5
Blast-related shrapnel	16	14.7
Others	2	1.8
AAST grade: small bowel (n = 75)		
Grade II	41	54.7
Grade III	33	44.0
Grade V	1	1.3
AAST grade: colorectal (n = 57)		
Grade II	33	57.9
Grade III	14	24.6
Grade IV	10	17.5
Surgical management: small bowel (n = 75)		
Primary repair	46	61.3
Resection and anastomosis	29	38.7
Surgical management: colon (n = 61)		
Primary repair	32	52.5
Resection and anastomosis	25	41.0
Stoma formation	4	6.6

Among small-bowel injuries, the jejunum was the most frequently injured segment (n = 60, 55.0%); among colorectal injuries, the transverse colon was most often involved (n = 26, 23.9%). AAST grade II injuries were the most common in both small bowel (n = 41, 54.7%) and colorectal (n = 33, 57.9%) injuries; grade IV injuries were observed only in colorectal injuries (n = 10, 17.5%). Injury severity was strongly associated with the surgical strategy adopted (small bowel: χ² = 161.90, p < 0.001; colon: χ² = 193.39, p < 0.001). Associated injuries were present in 71 patients (65.1%); the most frequent intra-abdominal associated injuries were retroperitoneal hematomas (n = 17, 15.6%) and stomach injury (n = 13, 11.9%); chest trauma (n = 21, 19.3%) and diaphragmatic injury (n = 18, 16.5%) were the most frequent extra-abdominal injuries.

Timing of enteral nutrition and group comparability

Ninety-four patients (86.2%) received EEN within 48 hours of surgery, while 15 patients (13.8%) received DEN. Baseline characteristics did not differ significantly between groups (Table 2). However, the DEN group had numerically higher rates of combined small-bowel and colon injuries (n = 6/15, 40.0% vs. n = 16/94, 17.0%), peritoneal contamination (n = 14/15, 93.3% vs. n = 68/94, 72.3%), and shock on admission (n = 7/15, 46.7% vs. n = 27/94, 28.7%).

**Table 2:** Baseline characteristics of patients with penetrating bowel injuries stratified by timing of postoperative enteral nutrition. Continuous variables compared by Mann-Whitney U test; categorical variables by χ² or Fisher's exact test. EEN: early enteral nutrition (≤48 h postoperatively); DEN: delayed enteral nutrition (>48 h); IQR: interquartile range; AAST: American Association for the Surgery of Trauma

Characteristic	EEN (n = 94)	DEN (n = 15)	p-value
Age (years), median (IQR)	28 (23-35)	24 (20-38)	0.555
Male, n (%)	89 (94.7)	13 (86.7)	0.247
Injury pattern, n (%)			0.151
Small bowel only	47 (50.0)	6 (40.0)	-
Colon only	31 (33.0)	3 (20.0)	-
Combined	16 (17.0)	6 (40.0)	-
Peritoneal contamination, n (%)	68 (72.3)	14 (93.3)	0.110
Time to surgery < 6 h, n (%)	92 (97.9)	15 (100)	1.000
AAST high grade (III-V), n (%)	41 (43.4)	8 (53.3)	0.482
Shock on admission, n (%)	27 (28.7)	7 (46.7)	0.229

Gastrointestinal recovery

Return of bowel sounds was significantly earlier in the EEN group (median 1 vs. 2 days; p = 0.021). The first passage of flatus occurred a median of three days after surgery in the EEN group versus four days in the DEN group (p = 0.091). Abdominal distention (n = 27/94, 28.7% vs. n = 7/15, 46.7%; p = 0.229) and vomiting (n = 9/94, 9.6% vs. n = 4/15, 26.7%; p = 0.079) were less frequent in the EEN group but did not reach statistical significance.

Postoperative complications

Detailed comparisons of postoperative complications are shown in Table 3. The overall complication rate was significantly lower in the EEN group than in the DEN group (n = 20/94, 21.3% vs. n = 9/15, 60.0%; risk ratio (RR) 0.36, 95% CI 0.20-0.63; p = 0.003). SSI occurred in n = 3/94, 3.2% vs. n = 7/15, 46.7%; RR 0.07, 95% CI 0.02-0.25; p < 0.001; fascial dehiscence in n = 1/94, 1.1% vs. n = 4/15, 26.7%; RR 0.04, 95% CI 0.01-0.33; p = 0.001. Anastomotic leak (n = 0/94, 0% vs. n = 3/15, 20.0%; p = 0.002), intra-abdominal abscess (n = 0/94, 0% vs. n = 1/15, 6.7%; p < 0.001), enterocutaneous fistula (n = 0/94, 0% vs. n = 2/15, 13.3%; p = 0.018), AKI (n = 0/94, 0% vs. n = 3/15, 20.0%; p = 0.002), septic shock (n = 0/94, 0% vs. n = 2/15, 13.3%; p = 0.018), sepsis (n = 1/94, 1.1% vs. n = 2/15, 13.3%; p = 0.049), and in-hospital mortality (n = 0/94, 0% vs. n = 3/15, 20.0%; p = 0.002) were all less frequent in the EEN group. Pneumonia (n = 16/94, 16.8% vs. n = 6/15, 40.0%; p = 0.076) and bowel obstruction (n = 6/94, 6.4% vs. n = 6/15, 20.0%; p = 0.107) were numerically less frequent with EEN but did not reach statistical significance.

**Table 3:** Postoperative complications, gastrointestinal recovery, and hospital-resource utilization by timing of enteral nutrition. Categorical variables compared by Fisher's exact test (small expected counts) or χ²; continuous variables by Mann-Whitney U test. RRs are not provided for outcomes with zero events in the EEN group. * indicates statistical significance (p < 0.05). EEN: early enteral nutrition (≤48 h); DEN: delayed enteral nutrition (>48 h); IQR: interquartile range; LOS: length of stay; ICU: intensive care unit; RR: risk ratio; CI: confidence interval

Outcome	EEN (n = 94)	DEN (n = 15)	p-value	RR (95% CI)
Bowel sounds (days), median (IQR)	1 (1-2)	2 (1-3)	0.021*	-
First flatus (days), median (IQR)	3 (2-4)	4 (3-5)	0.091	-
Vomiting, n (%)	9 (9.6)	4 (26.7)	0.079	-
Abdominal distention, n (%)	27 (28.7)	7 (46.7)	0.229	-
Overall complications, n (%)	20 (21.3)	9 (60.0)	0.003*	0.36 (0.20-0.63)
Surgical site infection, n (%)	3 (3.2)	7 (46.7)	<0.001*	0.07 (0.02-0.25)
Fascial dehiscence, n (%)	1 (1.1)	4 (26.7)	0.001*	0.04 (0.01-0.33)
Anastomotic leak, n (%)	0 (0.0)	3 (20.0)	0.002*	-
Intra-abdominal abscess, n (%)	0 (0.0)	1 (6.7)	<0.001*	-
Enterocutaneous fistula, n (%)	0 (0.0)	2 (13.3)	0.018*	-
Bowel obstruction, n (%)	6 (6.4)	3 (20.0)	0.107	-
Sepsis, n (%)	1 (1.1)	2 (13.3)	0.049	0.08 (0.01-0.83)
Septic shock, n (%)	0 (0.0)	2 (13.3)	0.018*	-
Acute kidney injury, n (%)	0 (0.0)	3 (20.0)	0.002*	-
Pneumonia, n (%)	16 (16.8)	6 (40.0)	0.076	-
In-hospital mortality, n (%)	0 (0.0)	3 (20.0)	0.002	-
Hospital LOS (days), median (IQR)	5 (4-7)	11 (8-28)	<0.001*	-
ICU LOS (days), median (IQR)	0 (0-2)	4 (1-6)	<0.001*	-
ICU admission, n (%)	33 (34.0)	12 (80.0)	0.001	-
Reoperation, n (%)	8 (8.4)	7 (46.7)	0.001	-

Hospital resource utilization

Patients receiving EEN had a markedly shorter median hospital stay (5 days, IQR 4-7 vs. 11 days, IQR 8-28; p < 0.001) and a shorter median ICU stay (0 days vs. 4 days; p < 0.001). ICU admission was less common in the EEN group (n = 33/94, 34% vs. n = 12/15, 80%; p = 0.001), as was the need for reoperation (n = 8/94, 8.4% vs. n = 7/15, 46.7%; p = 0.001).

Independent predictors of complications

In the exploratory multivariable logistic regression model, DEN after 48 hours was the only covariate that reached statistical significance for postoperative complications (adjusted OR 3.98, 95% CI 1.20-13.23; p = 0.024). High AAST grade, peritoneal contamination, and associated injuries were associated with higher point estimates but did not reach statistical significance. Because the DEN group was small and the number of events was limited, this finding should be interpreted as an adjusted association rather than proof of an independent causal effect (Table 4). The model was statistically significant (omnibus χ² = 15.94; p = 0.003), with an adequate fit (Hosmer-Lemeshow χ² = 5.43; p = 0.608), and explained 19.8% of the variance in complications (Nagelkerke R² = 0.198).

**Table 4:** Multivariable logistic regression for independent predictors of postoperative complications. Model omnibus χ² = 15.94, p = 0.003; Hosmer-Lemeshow goodness-of-fit χ² = 5.43, p = 0.608; Nagelkerke R² = 0.198. Variables with p < 0.20 on univariate analysis or with prior clinical relevance were entered into the multivariable model. * indicates statistical significance (p < 0.05). OR: odds ratio; CI: confidence interval; AAST: American Association for the Surgery of Trauma

Variable	Adjusted OR	95% CI	p-value
Delayed enteral nutrition (>48 h)	3.98	1.20-13.23	0.024*
AAST high grade (III-V)	1.74	0.67-4.54	0.254
Peritoneal contamination	2.74	0.67-10.88	0.152
Associated injuries	2.41	0.83-7.10	0.106
Constant	0.03	-	<0.001*

## Discussion

In this retrospective cohort of 109 adults undergoing operative repair of PBI during the Yemeni civilian war, EEN within 48 hours of surgery was associated with significantly faster gastrointestinal recovery, fewer postoperative complications, shorter hospital and ICU stays, fewer reoperations, and lower mortality compared with delayed feeding. Critically, on multivariable analysis, DEN remained the only independent predictor of postoperative complications. To our knowledge, this is one of the first cohort studies to evaluate EEN after PBI repair in a Yemeni conflict-zone hospital.

The demographic profile of our cohort-predominantly young men injured by gunshot wounds-closely mirrors series from comparable conflicts in Afghanistan, Iraq, and Syria [15-18]. The dominance of small-bowel injuries (51.4% isolated, 80% of which had multiple perforations) and the relative concentration of colonic injuries in the transverse and right colon are consistent with the anatomical exposure and mobility of these segments [19-21]. Our surgical management pattern-predominantly primary repair for low-grade injuries and resection-anastomosis for higher grades, with stoma formation reserved primarily for severely contaminated or unstable left colon injuries-aligns with the World Society of Emergency Surgery (WSES) guidelines on bowel trauma [7] and with multicenter data, including the AAST prospective study by Demetriades et al. and the recent Eastern Association for the Surgery of Trauma (EAST) multicenter trial by Fitzgerald et al. [14,22,23].

Our principal findings extend a large body of evidence supporting EEN. The reductions in overall complications (RR 0.36) and infectious complications, particularly SSI (RR 0.07), in the EEN group are consistent with systematic reviews and meta-analyses across abdominal surgery populations [8,24]. The biologically plausible mechanisms-preservation of mucosal barrier function, attenuation of bacterial translocation, immune modulation, and direct support of anastomotic collagen synthesis-have been demonstrated in clinical and experimental studies [3,4,25]. The absence of anastomotic leaks and enterocutaneous fistulas in the EEN group challenges the long-held surgical concern that early feeding stresses fresh anastomoses; rather, our results align with meta-analyses suggesting that early oral feeding reduces anastomotic leakage after gastrointestinal surgery [26,27]. The mortality difference (0% vs. 20%) is striking but is consistent with a meta-analysis of randomized trials in trauma patients requiring intensive care, which reported a mortality reduction with EEN (OR 0.20, 95% CI 0.04-0.91) [28]. These findings contrast with older guidance, notably the 2004 EAST practice-management recommendations, which did not demonstrate an outcome advantage of feeding within 24 hours over 72 hours [29].

The most important interpretive issue is confounding by indication. Patients in whom feeding was delayed may have been perceived by surgeons as being at higher risk because of more severe injury patterns, contamination, shock, physiologic instability, or concern regarding anastomotic safety. In this cohort, the DEN group had numerically higher rates of combined small-bowel and colon injury, peritoneal contamination, and shock on admission, although these differences did not reach statistical significance. Multivariable adjustment for high AAST grade, contamination, and associated injuries reduced but cannot eliminate this bias. Therefore, our findings should be interpreted as a strong association between delayed feeding and worse outcomes, not definitive evidence that delayed feeding directly caused those outcomes.

The observed differences in hospital and ICU length of stay have practical importance in a conflict-zone hospital. A median hospital stay of 5 days with EEN compared with 11 days with DEN, and a median ICU stay of 0 versus 4 days, suggests that earlier feeding may contribute to faster turnover of scarce surgical and ICU beds when clinically feasible. In mass-casualty or resource-constrained settings, even modest reductions in bed-days can expand operative capacity, decrease ICU congestion, and improve the ability to absorb subsequent trauma admissions. These operational implications are particularly relevant where commercial nutritional formulas, laboratory monitoring, and ICU staffing are intermittently constrained [30].

Limitations

Several limitations should be emphasized. First, this was a retrospective single-center study from a conflict-zone hospital, limiting external validity and making the analysis vulnerable to selection bias, missing data, and residual confounding. Second, confounding by indication is a major concern: delayed feeding was not randomized and likely reflected attending surgeons’ concern in patients perceived to be at higher risk. Although we adjusted for high AAST grade, peritoneal contamination, and associated injuries, the DEN group was small and numerically more severely injured, and unmeasured physiologic factors may still explain part of the association. Third, patients who died within 24 hours were excluded because they could not be classified meaningfully by postoperative feeding exposure. This improves exposure classification but introduces potential survival bias by excluding the most severely injured early deaths. Fourth, no standardized written feeding protocol existed during the study period; feeding initiation, interruption, and advancement were based on bedside clinical assessment and attending surgeon judgment. This limits reproducibility and makes it difficult to separate the effect of feeding timing from the clinical reasoning that led surgeons to delay feeding. Fifth, the small DEN group and the limited number of complication events reduce statistical power and may make the multivariable logistic regression estimates unstable. The adjusted OR for DEN had a wide CI and should be interpreted as exploratory. Sixth, surgeon identity, surgeon-level practice variation, antibiotic regimen and duration, caloric/protein delivery, and objective nutritional markers such as prealbumin were not consistently available for analysis. These missing variables precluded dose-response analysis and surgeon-level sensitivity analysis. Seventh, long-term follow-up beyond the index admission and 30-day readmission window was unavailable. Finally, because multiple outcomes were compared in a small cohort, statistically significant secondary findings should be interpreted in the context of biological plausibility and consistency with prior literature rather than as definitive causal estimates.

Future studies should prospectively evaluate EEN after PBI repair across multiple conflict-affected and resource-limited hospitals. Ideally, these studies should use standardized feeding initiation and advancement criteria, predefined stopping rules for intolerance, uniform antibiotic protocols where feasible, structured injury-severity measures, surgeon-level variables, and objective nutritional markers such as caloric/protein delivery, albumin, and prealbumin. Randomized or pragmatic stepped-wedge designs would provide stronger evidence while respecting the operational constraints of emergency trauma care.

## Conclusions

In adults undergoing operative repair of PBIs in a Yemeni civil-war setting, initiation of enteral nutrition within 48 hours was feasible and was associated with faster gastrointestinal recovery, fewer postoperative complications, shorter hospital and ICU lengths of stay, and lower mortality than delayed feeding. Because this was a retrospective single-center cohort with a small delayed-feeding group, the findings should be interpreted as hypothesis-generating associations rather than proof of causality. These data support considering EEN when clinically feasible after adequate source control and stabilization in resource-limited trauma settings, and they highlight the need for prospective multicenter studies using standardized feeding protocols.

## Appendices

Standardized data collection form for retrospective chart review

**Table 5:** Patient demographics and medical history. Variables abstracted from the patient’s medical record at the time of index hospitalization.

Variable	Response options
A. Administrative and demographic variables	
Enrolling center	Al-Safwa Hospital
Assigned patient ID	Alphanumeric (free text)
Age	Years
Sex	Male/female
Address-governorate and district	Free text
Occupation	Free text
Tobacco use	Never/current/former
B. Comorbidities	
Diabetes mellitus	No/yes-without end-organ damage/yes-with end-organ damage
Diabetes-insulin-dependent	No/yes
Hypertension	No/yes
Ischemic heart disease	No/yes
Congestive heart failure	No/yes
Atrial fibrillation	No/yes
Peripheral vascular disease	No/yes
Cerebrovascular accident (CVA)	No/CVA without hemiplegia/CVA with hemiplegia or paraplegia
Cancer-type	None/lymphoma/leukemia/solid tumor (specify)
Cancer-metastatic disease	No/yes
Cancer-currently receiving chemotherapy	No/yes
Chronic kidney disease	No/yes
Chronic pulmonary disease	No/yes
Liver cirrhosis	No/yes
HIV/AIDS	No/yes
Prior abdominal surgery	No/yes
Prior abdominal surgery-type	Stomach/gastric bypass/pancreas/spleen/small bowel/colon/liver/other (specify)
C. Medication history	
Anticoagulant or antiplatelet therapy	None/warfarin/aspirin/other (specify)
Corticosteroid use	No/yes
Other immunosuppressant	No/yes (specify agent)

**Table 6:** Injury characteristics and admission variables. Laboratory values represent the worst measurement recorded in the 24 hours prior to the index operation.

Variable	Response options
A. Injury characteristics	
Date of injury	MM/DD/YYYY
Time of injury	HH:MM (24-hour clock)
Mechanism of penetrating injury	Gunshot wound/shrapnel or blast injury/stab wound/other (specify)
Intent or manner of injury	War-related/assault or homicide/self-inflicted/unintentional occupational/unintentional non-occupational/unspecified
Major associated injuries-head or neck	No/yes (specify)
Major associated injuries-face	No/yes (specify)
Major associated injuries-chest	No/yes (specify)
Major associated injuries-abdomen	No/yes (specify)
Major associated injuries-extremities or bony pelvis	No/yes (specify)
B. Admission vital signs	
Date of admission	MM/DD/YYYY
Time of admission	HH:MM (24-hour clock)
Systolic blood pressure (SBP) on admission	mmHg
Diastolic blood pressure on admission	mmHg
Heart rate on admission	Beats per minute
Respiratory rate on admission	Breaths per minute
Temperature on admission	Degrees Celsius
Shock on admission (SBP < 90 mmHg)	No/yes
C. Admission laboratory values (worst value in 24 hours prior to index operation)	
Hemoglobin	g/dL
Hematocrit	Percentage
White blood cell count	×10³/μL
Platelet count	×10³/μL
Serum creatinine	mg/dL-enter 999 if on renal replacement therapy
Serum albumin	g/dL
Total serum bilirubin	mg/dL

**Table 7:** Operative variables-index operation. Bowel segment subvariables are recorded for every segment. Enter N/A for all subvariables of segments not injured.

Variable	Response options
A. General operative details	
Date of operation	MM/DD/YYYY
Time of operation	HH:MM (24-hour clock)
Time from injury to laparotomy	Hours
Delay category to operative intervention	<6 h/6-12 h/12-24 h/>24 h
Duration of operation	Minutes-enter 999 if not applicable
Primary surgeon qualification	Experience only/Master's degree/Board-certified/Fellowship-trained/PhD/MD/full consultant
Primary surgeon's years of experience	Years
Trainee participation	No/yes
B. Small bowel injury-duodenum first part (D1)	
D1-injured	No/yes
D1-injury type	Single perforation/multiple perforations/transection/N/A
D1-management	Primary suture repair/resection and anastomosis/primary repair details unavailable/N/A
D1-anastomosis location	Jejuno-jejunal/ileo-jejunal/ileo-ileal/ileocolic/other (specify)/N/A
D1-anastomosis orientation	End-to-end/end-to-side/side-to-side/N/A
D1-anastomosis method	Handsewn single-layer/handsewn two-layer/layers unknown/N/A
C. Small bowel injury-duodenum second part (D2)	
D2-injured	No/yes
D2-injury type	Single perforation/multiple perforations/transection/N/A
D2-management	Primary suture repair/resection and anastomosis/primary repair details unavailable/N/A
D2-anastomosis location	Jejuno-jejunal/ileo-jejunal/ileo-ileal/ileocolic/other (specify)/N/A
D2-anastomosis orientation	End-to-end/end-to-side/side-to-side/N/A
D2-anastomosis method	Handsewn single-layer/handsewn two-layer/layers unknown/N/A
D. Small bowel injury-duodenum third part (D3)	
D3-injured	No/yes
D3-injury type	Single perforation/multiple perforations/transection/N/A
D3-management	Primary suture repair/resection and anastomosis/primary repair details unavailable/N/A
D3-anastomosis location	Jejuno-jejunal/ileo-jejunal/ileo-ileal/ileocolic/other (specify)/N/A
D3-anastomosis orientation	End-to-end/end-to-side/side-to-side/N/A
D3-anastomosis method	Handsewn single-layer/handsewn two-layer/layers unknown/N/A
E. Small bowel injury-duodenum fourth part (D4)	
D4-injured	No/yes
D4-injury type	Single perforation/multiple perforations/transection/N/A
D4-management	Primary suture repair/resection and anastomosis/primary repair details unavailable/N/A
D4-anastomosis location	Jejuno-jejunal/ileo-jejunal/ileo-ileal/ileocolic/other (specify)/N/A
D4-anastomosis orientation	End-to-end/end-to-side/side-to-side/N/A
D4-anastomosis method	Handsewn single-layer/handsewn two-layer/layers unknown/N/A
F. Small bowel injury-jejunum	
Jejunum-injured	No/yes
Jejunum-injury type	Single perforation/multiple perforations/transection/N/A
Jejunum-management	Primary suture repair/resection and anastomosis/primary repair details unavailable/N/A
Jejunum-resection specimen location	Localized debridement only/proximal jejunum/distal jejunum/N/A
Jejunum-length of resected bowel	cm-N/A if no resection
Jejunum-anastomosis location	Jejuno-jejunal/ileo-jejunal/ileo-ileal/ileocolic/other (specify)/N/A
Jejunum-anastomosis orientation	End-to-end/end-to-side/side-to-side/N/A
Jejunum-anastomosis method	Handsewn single-layer/handsewn two-layer/layers unknown/N/A
G. Small bowel injury-ileum	
Ileum-injured	No/yes
Ileum-injury type	Single perforation/multiple perforations/transection/N/A
Ileum-management	Primary suture repair/resection and anastomosis/primary repair details unavailable/N/A
Ileum-resection specimen location	Proximal ileum/distal ileum/distal ileum with ileocecal valve/localized debridement only/N/A
Ileum-length of resected bowel	cm-N/A if no resection
Ileum-anastomosis location	Jejuno-jejunal/ileo-jejunal/ileo-ileal/ileocolic/other (specify)/N/A
Ileum-anastomosis orientation	End-to-end/end-to-side/side-to-side/N/A
Ileum-anastomosis method	Handsewn single-layer/handsewn two-layer/layers unknown/N/A
H. Large bowel injury-cecum	
Cecum-injured	No/yes
Cecum-injury type	Single perforation/multiple perforations/transection/N/A
Cecum-management	Primary suture repair/resection and anastomosis/primary repair details unavailable/N/A
Cecum-resection specimen	Localized debridement only/ileocolic resection/right hemicolectomy/N/A
Cecum-length of resected bowel	cm-N/A if no resection
Cecum-anastomosis location	Ileocolic/colo-colonic/colorectal/ileorectal/other (specify)/N/A
Cecum-anastomosis orientation	End-to-end/end-to-side/side-to-side/N/A
Cecum-anastomosis method	Handsewn single-layer/handsewn two-layer/layers unknown/N/A
I. Large bowel injury-ascending colon	
Ascending colon-injured	No/yes
Ascending colon-injury type	Single perforation/multiple perforations/transection/N/A
Ascending colon-management	Primary suture repair/resection and anastomosis/primary repair details unavailable/N/A
Ascending colon-resection specimen	Localized debridement only/right hemicolectomy/extended right hemicolectomy/transverse colectomy/N/A
Ascending colon-length of resected bowel	cm-N/A if no resection
Ascending colon-anastomosis location	Ileocolic/colo-colonic/colorectal/ileorectal/other (specify)/N/A
Ascending colon-anastomosis orientation	End-to-end/end-to-side/side-to-side/N/A
Ascending colon-anastomosis method	Handsewn single-layer/handsewn two-layer/layers unknown/N/A
J. Large bowel injury-transverse colon	
Transverse colon-injured	No/yes
Transverse colon-injury type	Single perforation/multiple perforations/transection/N/A
Transverse colon-management	Primary suture repair/resection and anastomosis/primary repair details unavailable/N/A
Transverse colon-resection specimen	Localized debridement only/transverse colectomy/extended right hemicolectomy/left hemicolectomy/N/A
Transverse colon-length of resected bowel	cm-N/A if no resection
Transverse colon-anastomosis location	Ileocolic/colo-colonic/colorectal/ileorectal/other (specify)/N/A
Transverse colon-anastomosis orientation	End-to-end/end-to-side/side-to-side/N/A
Transverse colon-anastomosis method	Handsewn single-layer/handsewn two-layer/layers unknown/N/A
K. Large bowel injury-descending colon	
Descending colon-injured	No/yes
Descending colon-injury type	Single perforation/multiple perforations/transection/N/A
Descending colon-management	Primary suture repair/resection and anastomosis/primary repair details unavailable/N/A
Descending colon-resection specimen	Localized debridement only/left hemicolectomy/sigmoid colectomy/N/A
Descending colon-length of resected bowel	cm-N/A if no resection
Descending colon-anastomosis location	Ileocolic/colo-colonic/colorectal/ileorectal/other (specify)/N/A
Descending colon-anastomosis orientation	End-to-end/end-to-side/side-to-side/N/A
Descending colon-anastomosis method	Handsewn single-layer/handsewn two-layer/layers unknown/N/A
L. Large bowel injury-sigmoid colon	
Sigmoid colon-injured	No/yes
Sigmoid colon-injury type	Single perforation/multiple perforations/transection/N/A
Sigmoid colon-management	Primary suture repair/resection and anastomosis/primary repair details unavailable/N/A
Sigmoid colon-resection specimen	Localized debridement only/sigmoid colectomy/anterior resection/subtotal colectomy/N/A
Sigmoid colon-length of resected bowel	cm-N/A if no resection
Sigmoid colon-anastomosis location	Ileocolic/colo-colonic/colorectal/ileorectal/other (specify)/N/A
Sigmoid colon-anastomosis orientation	End-to-end/end-to-side/side-to-side/N/A
Sigmoid colon-anastomosis method	Handsewn single-layer/handsewn two-layer/layers unknown/N/A
M. Large bowel injury-intraperitoneal rectum	
Intraperitoneal rectum-injured	No/yes
Intraperitoneal rectum-injury type	Single perforation/multiple perforations/transection/N/A
Intraperitoneal rectum-management	Primary suture repair/resection and anastomosis/primary repair details unavailable/N/A
Intraperitoneal rectum-resection specimen	Localized debridement only/anterior resection/subtotal colectomy/total abdominal colectomy/N/A
Intraperitoneal rectum-length of resected bowel	cm-N/A if no resection
Intraperitoneal rectum-anastomosis location	Ileocolic/colo-colonic/colorectal/ileorectal/other (specify)/N/A
Intraperitoneal rectum-anastomosis orientation	End-to-end/end-to-side/side-to-side/N/A
Intraperitoneal rectum-anastomosis method	Handsewn single-layer/handsewn two-layer/layers unknown/N/A
N. Large bowel injury-extraperitoneal rectum	
Extraperitoneal rectum-injured	No/yes
Extraperitoneal rectum-injury type	Single perforation/multiple perforations/transection/N/A
Extraperitoneal rectum-management	Primary suture repair/resection and anastomosis/primary repair details unavailable/N/A
Extraperitoneal rectum-resection specimen	Localized debridement only/anterior resection/subtotal colectomy/total abdominal colectomy/N/A
Extraperitoneal rectum-length of resected bowel	cm-N/A if no resection
Extraperitoneal rectum-anastomosis location	Ileocolic/colo-colonic/colorectal/ileorectal/other (specify)/N/A
Extraperitoneal rectum-anastomosis orientation	End-to-end/end-to-side/side-to-side/N/A
Extraperitoneal rectum-anastomosis method	Handsewn single-layer/handsewn two-layer/layers unknown/N/A
O. Large bowel injury-colon (site unspecified)	
Colon unspecified-injured	No/yes
Colon unspecified-injury type	Single perforation/multiple perforations/transection/N/A
Colon unspecified-management	Primary suture repair/resection and anastomosis/primary repair details unavailable/N/A
Colon unspecified-resection specimen	Localized debridement only/right hemicolectomy/left hemicolectomy/sigmoid colectomy/other/N/A
Colon unspecified-length of resected bowel	cm-N/A if no resection
Colon unspecified-anastomosis location	Ileocolic/colo-colonic/colorectal/ileorectal/other (specify)/N/A
Colon unspecified-anastomosis orientation	End-to-end/end-to-side/side-to-side/N/A
Colon unspecified-anastomosis method	Handsewn single-layer/handsewn two-layer/layers unknown/N/A
P. Other intraoperative findings and interventions	
Peritoneal contamination	No/yes/N/A
Retained intra-abdominal missile or foreign body	No/yes/N/A
Other intra-abdominal injury-abdominal vascular	No/yes
Other intra-abdominal injury-retroperitoneal hematoma	No/yes
Other intra-abdominal injury-diaphragm	No/yes
Other intra-abdominal injury-liver laceration	No/yes
Other intra-abdominal injury-splenic laceration	No/yes
Other intra-abdominal injury-renal laceration	No/yes
Other intra-abdominal injury-gastric	No/yes
Other intra-abdominal injury-pancreatic	No/yes
Other intra-abdominal injury-bladder	No/yes
Other intra-abdominal injury-ureteric	No/yes
Additional intervention-abdominal packing	No/yes
Additional intervention-vascular repair	No/yes (specify location and type)
Additional intervention-diaphragm repair	No/yes
Additional intervention-hepatorrhaphy	No/yes
Additional intervention-hepatic resection	No/yes
Additional intervention-splenorrhaphy	No/yes
Additional intervention-splenectomy	No/yes
Additional intervention-nephrorrhaphy	No/yes
Additional intervention-nephrectomy	No/yes
Additional intervention-gastric repair or resection	No/yes
Additional intervention-pancreatic repair	No/yes
Additional intervention-bladder repair	No/yes
Additional intervention-ureteric repair	No/yes
Additional intervention-posterolateral thoracotomy	No/yes
Additional intervention-anterolateral thoracotomy	No/yes
Peritoneal toilet performed	No/yes/N/A
Abdominal drain(s) inserted at index operation	No/yes
Fascial closure achieved	No/yes
Skin closure at time of fascial closure	No/yes
Extra-abdominal procedure(s) at index operation	None/yes (specify)
Intraoperative hypotension (single systolic blood pressure (SBP) < 90 mmHg)	No/yes
Intraoperative vasopressor infusion required	No/yes

**Table 8:** Nutritional management, postoperative clinical evaluation, and complications. Record the postoperative day (POD) of first occurrence for each event. Record N/A for subvariables that do not apply.

Variable	Response options
A. Postoperative clinical evaluation (record POD of first occurrence)	
Abdominal distension	No/yes-POD: ___
Nausea and vomiting	No/yes-POD: ___
First passage of flatus or stool	No/yes-POD: ___
B. Nutritional management	
Early enteral nutrition initiated	No/yes-POD started: ___
Route of enteral nutrition	Oral/nasogastric tube/gastrostomy/jejunostomy/N/A
Formula or type of enteral feed	Free text-N/A if not initiated
Parenteral nutrition required	No/yes-POD started: ___
Type or formula of parenteral nutrition	Free text-N/A if not required
C. Surgical site and intra-abdominal complications (record POD of first discovery)	
Postoperative hemorrhage requiring transfusion	No/yes-POD: ___
Postoperative hemorrhage requiring reoperation	No/yes-POD: ___
Fascial dehiscence	No/yes-POD: ___
Anastomotic or suture-line leak or dehiscence	No/yes-POD: ___
Enterocutaneous or enteroatmospheric fistula	No/yes-POD: ___
Bowel obstruction requiring operative intervention	No/yes-POD: ___
Superficial surgical site infection	No/yes-POD: ___
Deep incisional surgical site infection	No/yes-POD: ___
Intra-abdominal abscess or organ-space SSI	No/yes-POD: ___
Percutaneous drain placed for complication	No/yes-POD: ___
Antibiotic initiation or modification for complication	No/yes
D. Systemic complications (record POD of first discovery)	
Acute kidney injury (AKI)	No/yes-POD: ___
AKI-hemodialysis required	No/yes/N/A
AKI-peak creatinine	mg/dL-N/A if no AKI
Anemia requiring transfusion	No/yes-POD: ___
Urinary tract infection	N/A/no/yes-POD: ___
Sepsis	No/yes-POD: ___
Septic shock	No/yes-POD: ___
Multiple organ failure	No/yes-POD: ___
Acute respiratory distress syndrome	No/yes-POD: ___
Pneumonia	No/yes-POD: ___
Myocardial infarction	No/yes-POD: ___
Cerebrovascular accident	No/yes-POD: ___
Deep vein thrombosis or pulmonary embolism	No/yes-POD: ___
Postoperative paralytic ileus	No/yes-POD: ___
Postoperative paralytic ileus-duration	Days-N/A if no ileus
Other complication (specify)	No/yes-POD: ___-specify: ___
E. Unplanned return to operating room	
Unplanned return to operating room	No/yes
Total number of unplanned returns	Count-N/A if none
Return 1-indication	Free text-N/A if not applicable
Return 1-main intraoperative finding	Free text-N/A if not applicable
Return 1-procedure(s) performed	Free text-N/A if not applicable
Return 1-POD	POD-N/A if not applicable
Return 2-indication	Free text-N/A if not applicable
Return 2-main intraoperative finding	Free text-N/A if not applicable
Return 2-procedure(s) performed	Free text-N/A if not applicable
Return 2-POD	POD-N/A if not applicable
Return 3-indication	Free text-N/A if not applicable
Return 3-main intraoperative finding	Free text-N/A if not applicable
Return 3-procedure(s) performed	Free text-N/A if not applicable
Return 3-POD	POD-N/A if not applicable
Secondary intervention-laparoscopic	No/yes
Secondary intervention-open surgery	No/yes
Secondary intervention-hemostasis	No/yes
Secondary intervention-drainage of collection	No/yes
Secondary intervention-lysis of adhesions	No/yes
Secondary intervention-small bowel resection	No/yes
Secondary intervention-large bowel resection	No/yes
Secondary intervention-suture-line repair	No/yes
Secondary intervention-retention sutures	No/yes
Secondary intervention-stoma creation	No/yes (specify type)
Secondary intervention-missed injury management	No/yes
Secondary intervention-other	No/yes (specify)
Readmission within 30 days of discharge	No/yes
Readmission-reason	Free text-N/A if no readmission
Readmission-day after discharge	Days-N/A if no readmission

**Table 9:** Discharge and outcome parameters. Complete mortality subvariables only for patients who died during the index hospitalization; record N/A for all mortality fields if the patient was discharged alive.

Variable	Response options
A. Discharge variables	
Date of hospital discharge	MM/DD/YYYY
Time of hospital discharge	HH:MM (24-hour clock)
Total hospital length of stay	Days
Total intensive care unit (ICU) length of stay	Days-record 0 if not admitted to ICU
Total ventilator days	Days-record 0 if not mechanically ventilated
Discharge status	Alive/deceased in-hospital
B. In-hospital mortality (complete only if patient died during index hospitalization; otherwise, record N/A)	
Postoperative day (POD) of death	POD
Date of death	MM/DD/YYYY
Time of death	HH:MM (24-hour clock)
Location of death	Emergency department/operating room/surgical ICU/general ward/other (specify)
Death attributed to bowel injury	No/yes/unknown
Cause of death-exsanguination or hemorrhagic shock	No/yes
Cause of death-traumatic brain injury	No/yes
Cause of death-respiratory failure	No/yes
Cause of death-sepsis or multiple organ failure	No/yes
Cause of death-cardiovascular event	No/yes
Cause of death-pulmonary embolism	No/yes
Cause of death-other (specify)	No/yes (specify)
